# Drug Conjugation via Maleimide–Thiol Chemistry Does Not Affect Targeting Properties of Cysteine-Containing Anti-FGFR1 Peptibodies

**DOI:** 10.1021/acs.molpharmaceut.1c00946

**Published:** 2022-04-07

**Authors:** Karolina Jendryczko, Jakub Rzeszotko, Mateusz Adam Krzyscik, Anna Kocyła, Jakub Szymczyk, Jacek Otlewski, Anna Szlachcic

**Affiliations:** † Department of Protein Engineering, University of Wroclaw, Wroclaw 50-383, Poland; ‡ Department of Chemical Biology, University of Wroclaw, Wroclaw 50-383, Poland

**Keywords:** targeting peptides, cytotoxic conjugates, peptide−Fc fusions, peptibodies, targeted therapies, FGFR1

## Abstract

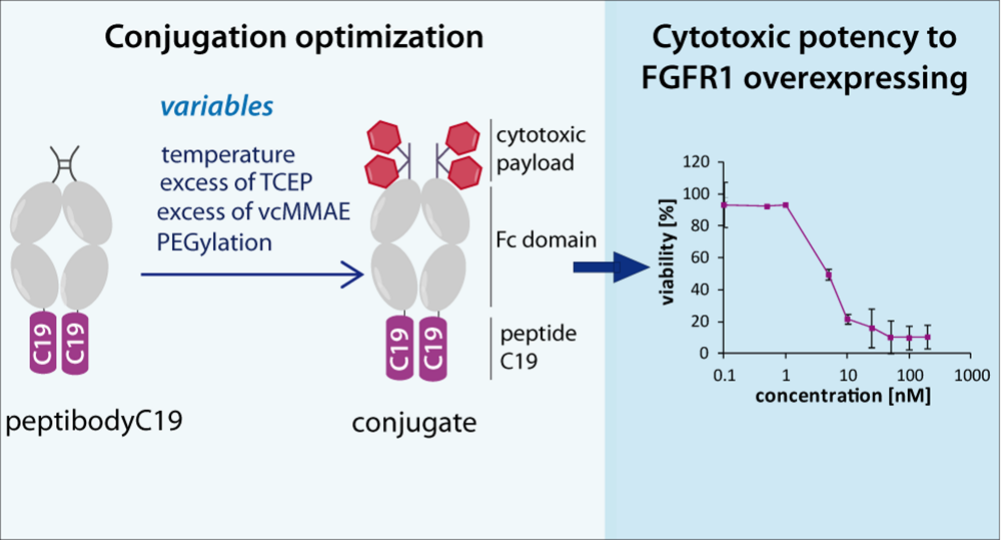

With a wide range of available cytotoxic therapeutics, the main focus of current cancer research is to deliver them specifically to the cancer cells, minimizing toxicity against healthy tissues. Targeted therapy utilizes different carriers for cytotoxic drugs, combining a targeting molecule, typically an antibody, and a highly toxic payload. For the effective delivery of such cytotoxic conjugates, a molecular target on the cancer cell is required. Various proteins are exclusively or abundantly expressed in cancer cells, making them a possible target for drug carriers. Fibroblast growth factor receptor 1 (FGFR1) overexpression has been reported in different types of cancer, but no FGFR1-targeting cytotoxic conjugate has been approved for therapy so far. In this study, the FGFR1-targeting peptide previously described in the literature was reformatted into a peptibody–peptide fusion with the fragment crystallizable (Fc) domain of IgG1. PeptibodyC19 can be effectively internalized into FGFR1-overexpressing cells and does not induce cells’ proliferation. The main challenge for its use as a cytotoxic conjugate is a cysteine residue located within the targeting peptide. A standard drug-conjugation strategy based on the maleimide–thiol reaction involves modification of cysteines within the Fc domain hinge region. Applied here, however, may easily result in the modification of the targeting peptide with the drug, limiting its affinity to the target and therefore the potential for specific drug delivery. To investigate if this is the case, we have performed conjugation reactions with different auristatin derivatives (PEGylated and unmodified) under various conditions. By controlling the reduction conditions and the type of cytotoxic payload, different numbers of cysteines were substituted, allowing us to avoid conjugating the drug to the targeting peptide, which could affect its binding to FGFR1. The optimized protocol with PEGylated auristatin yielded doubly substituted peptibodyC19, showing specific cytotoxicity toward the FGFR1-expressing lung cancer cells, with no effect on cells with low FGFR1 levels. Indeed, additional cysteine poses a risk of unwanted modification, but changes in the type of cytotoxic payload and reaction conditions allow the use of standard thiol–maleimide-based conjugation to achieve standard Fc hinge region cysteine modification, analogously to antibody–drug conjugates.

## Introduction

Classical chemotherapy used in cancer treatment displays high systemic toxicity. Currently, targeted therapies are rapidly emerging both in preclinical and clinical studies, with several approved treatments in the market, such as erdafitinib,^1^ imatinib,^2^ and rituximab.^3^ The rationale behind this type of therapy instead of traditional cancer treatment is reducing the side effects by increasing specificity and affecting only cells displaying cancerous characteristics.

Specific delivery of the therapeutic agent is the cornerstone of this approach, and multiple different types of molecules have been developed, the majority of which are monoclonal antibodies (mAbs) or mAb-based formats. Targeting molecules can directly affect cancer cells but can also be utilized as carriers for cytotoxic drugs. The most studied type of molecules used in this approach, with several examples already in clinical use, are antibody–drug conjugates (ADCs). They consist of a monoclonal antibody specific to a molecular target presented on cancer cells and a covalently attached cytotoxic drug. Several ADCs have been accepted for clinical use and show gratifying efficacy, such as brentuximab vedotin and trastuzumab emtansine.^4^

One of the most frequent methods for conjugating drugs with mAbs and fragment crystallizable (Fc)-fusion proteins take advantage of cysteine residues.^5^ After the reduction of interchain disulfide bonds, thiol groups can be utilized as attachment points for the payload. To make the drug available for connection to thiol groups, it can be functionalized with maleimide. This approach has been used to produce, e.g., an FDA-approved ADC, brentuximab vedotin.^6^ Other methods include lysine modification (ado-trastuzumab emtansine^7^), the introduction of unnatural amino acids,^8^ and enzymatic modification with sortase A or transglutaminases.^9^ The main advantages of the maleimide–thiol reaction are mild conditions (i.e., pH close to physiological and absence of dangerous additives), stability of thioether bonds, and irreversibility of this modification under reducing conditions. Moreover, contrary to the *N*-hydroxysuccinimide (NHS)-primary amine reaction, the maleimide–thiol reaction does not change the net charge of biomolecules.^10^

Many different molecular targets, with the potential to be used in targeted therapy, have been described so far. These include mainly proteins overexpressed or expressed exclusively in cancer cells, i.e., human epidermal growth factor receptor 2 (HER2), vascular endothelial growth factor receptor (VEGFR), or Bcr-Abl fusion protein.^11^ One of the cancer-related proteins is also fibroblast growth factors (FGFs) and their receptors (FGFRs). They are involved in numerous processes in an organism, such as cell proliferation and differentiation, embryonic development, angiogenesis, and wound healing.^12,13^

As FGFRs mediate many functions related to cell cycle and division, they pose a risk of inducing malignant transformation and are considered proto-oncogenes.^14^ Different mechanisms can contribute to aberrant FGF signaling in cancer and the type of disorder is usually coupled with types of both FGFR and cancer. Non-small cell lung cancer (NSCLC) poses a serious threat to humans in developed countries and is associated with various FGFR aberrations.^15^ Amplification of FGFR1 was found in patients with squamous cell carcinoma of the lung and its prevalence was estimated at 19%.^16,17^ Point mutations of FGFR3 are often found in multiple myeloma and bladder, cervical, and prostate cancers.^18−20^ Mutations cause uncontrolled activation of the receptor and can be localized in extracellular or transmembrane regions of the receptor, leading to its constant dimerization, as well as in its kinase domains.^21−23^ For these reasons, FGFRs have become therapeutic targets for various types of targeted therapies, utilizing different mechanisms of action.

The first group of therapeutics used in the treatment of cancers overexpressing FGFRs is small molecules inhibiting the activity of tyrosine kinase domains—tyrosine kinase inhibitors (TKIs).^24^ Despite promising results, the use of TKIs carries the risk of developing drug resistance in tumor cells. mAbs are considered universal therapeutic binders and a few of them are being developed for FGFR-targeted therapy. For now, there is one mAb specific for FGFR3 enrolled in clinical trials, vofatamab.^25^ Other antibody-related formats for FGFR targeting include ligand traps, such as FP-1039, a fusion protein combining the FGFR1 extracellular region with the Fc domain of IgG1 (immunoglobulin G1).^26^ Also, antibodies in the single-chain fragment variable (scFv) format, consisting of variable regions of heavy and light chains connected by a linker, can serve to sequester FGFs. scFv and scFv-Fc fusions designed to disrupt FGF1-dependent signaling successfully inhibited the growth of various cancer cell lines in vitro.^27,28^

**Figure 1:**
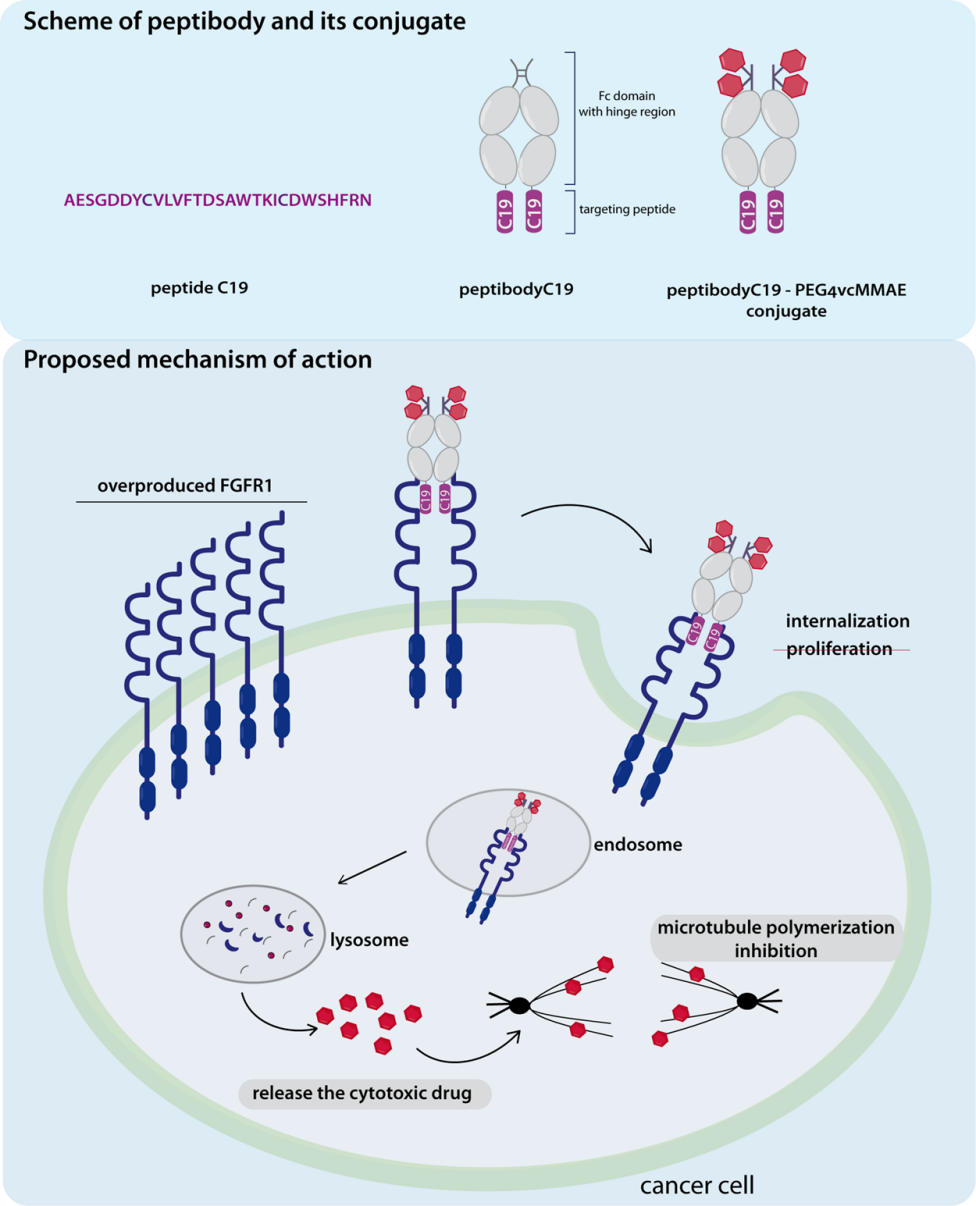
Proposed mechanism of action of the peptibody–drug conjugate. The peptibody is constructed based on an FGFR1-binding peptide, and the drug is covalently attached via cysteine modification in the Fc domain hinge region, analogous to ADCs. Once administered to cells overexpressing FGFR1, it is internalized and show toxicity after the drug is released.

In our recent studies, we have characterized FGFR1-targeting peptide–Fc fusions—peptibodies.^29,30^ In this approach, the properties of antibodies are combined with the flexibility in the design of targeting peptides.^31^ The function of the Fc fragment is mainly prolongation of the circulation time, which is a limiting factor in the case of free peptides.^32^ Fc fusions have a longer in vivo half-life primarily due to the neonatal Fc receptor (FcRn) salvage pathway,^33^ which mediates recycling of IgG and other proteins bearing the Fc domain back to the cell surface upon internalization. Additionally, the increased size of the molecule allows for avoidance of renal clearance, extending its half-life. The Fc domain is also responsible for activating immune response at the tumor site and is often utilized as a point of attachment for the cytotoxic payload.^34^ There are several peptibodies used in therapies (e.g., romiplostim for the treatment of immune thrombocytopenia^35^ and dulaglutide for type 2 diabetes treatment^36^) and many other molecules are being studied in clinical trials. As this drug format is relatively new, currently there are no peptibody–drug conjugates used in the cancer therapy. The proposed mechanism of peptibody–drug conjugate action is similar to ADCs and is presented in Figure 1.

Peptibody efficacy is dependent mostly on the targeting potential of an Fc-fused peptide. New peptidic binders can be identified using high throughput techniques, e.g., by phage display or cell-free display technologies,^37^ but the main limitation of peptides is their relatively lower target-binding affinities compared to the ones displayed by mAbs. Still, there are peptides with high-nanomolar affinities described for multiple receptors, including FGFRs. In 1999, a C19 peptide was found by phage display to show substantial FGFR1-binding and even strong FGFR agonist action when dimerized by the c-Jun leucine zipper.^38^ However, an Fc-fusion of the C19 peptide did not show such mitogenic potential, making it a suitable construct for potential targeting of FGFR-expressing cancer cells. Taking advantage of the high affinity of the C19 peptide toward FGFR1, we want to investigate if it can be used as a delivery vehicle for a highly cytotoxic drug, monomethyl auristatin E (MMAE). This task is not that straightforward, as the targeting peptide contains cysteine residues within its sequence; thus, an unwanted modification with the drug may occur not only within the Fc fragment, but also modification with the cytotoxic drug within the (relatively short) targeting sequence may lead to the weakening or loss of the interaction with the target (i.e., FGFR1).

Here, we present the optimization process (involving both conjugation reaction condition changes as well as functionalization of the drug itself) allowing for modification of the peptibody-Fc molecule in a controlled manner to obtain a desirable drug-to-protein ratio (DAR).

## Experimental Section

### Materials

#### Reagents

The chromatographic columns HiTrap MabSelect SuRe and HiTrap Desalting with Sephadex G-25 resin were obtained from GE Healthcare (UK). Reagents for microscopy CellLight Early Endosomes-RFP, BacMam 2.0 (#C10587), Zenon Alexa Fluor 488 Human IgG Labeling Kit (#Z25402), and NucBlue Live ReadyProbes Reagent (#R37605) were purchased from Thermo Fisher Scientific (Waltham, MA). Conjugation reagents tris(2-carboxyethyl) phosphine (TCEP) pH 7.0 (#646547) was obtained from Merck (Darmstadt, Germany), maleimidocaproyl-Val-Cit-PABC-monomethyl auristatin E (vcMMAE) (#HY-15575, MedChem Express).

#### Antibodies

The following primary antibodies were used: monoclonal anti-FGFR1 (#9740), monoclonal antiphospho-FGFR1 (#3476), polyclonal anti-p44/42 MAPK (Erk1/2) (#9102), and polyclonal antiphospho-p44/42 MAPK (Erk1/2) (#9101) from Cell Signaling (Danvers, MA). Monoclonal anti-γ-tubulin (#T6557) was provided by Sigma-Aldrich (St Louis, MO). Anti-human IgG Fc conjugated with HRP (horseradish peroxidase) was obtained from Abcam (#ab97225, Cambridge, UK). The following secondary antibodies were used for detection: anti-rabbit (#111-035-144) and anti-mouse (#115-035-003) from Jackson ImmunoResearch (Baltimore Pike, PA).

#### Cell Lines

CHO-S cells (Thermo Fisher Scientific, Waltham, MA) were cultured in PowerCHO medium (Lonza, Basel, Switzerland) supplemented with 8 mM l-glutamine and antibiotic mix (Biowest, Nuaillé, France). U2OS (human bone osteosarcoma epithelial cells), NIH 3T3 (mouse embryo fibroblasts), NCI-520 (lung squamous cell carcinoma), and NCI-H1581 (large cell lung carcinoma) were provided by ATCC (American Type Culture Collection). HCC95 (lung squamous cell carcinoma) were obtained from Drs. Minna and Gazdar from UT Southwestern Medical Center and cultured in RPMI 1640 (ATCC) with 10% FBS, antibiotic mix (100 U/mL penicillin and 100 μg/mL streptomycin) (Thermo Fisher Scientific, Waltham, MA), and sodium bicarbonate (Gibco, Waltham, MA). U2OS-FGFR1 (U2OS stably transfected with gene encoding FGFR1) were provided by Martyna Sochacka from our lab and cultured in DMEM HG with 10% fetal bovine serum, antibiotic mix (Biowest, Nuaillé, France), and 0.2 mg/mL geneticin (Thermo Fisher Scientific, Waltham, MA).

NCI-H1581 was cultured in RPMI 1640 (ATCC) with 10% FBS, antibiotic mix (100 U/mL penicillin and 100 μg/mL streptomycin) (Thermo Fisher Scientific, Waltham, MA), and sodium bicarbonate (Gibco, Waltham, MA). U2OS-FGFR1 (U2OS stably transfected with gene encoding FGFR1) were provided by Martyna Sochacka from our lab and cultured in DMEM HG with 10% fetal bovine serum antibiotic mix (Biowest, Nuaillé, France) and 0.2 mg/mL geneticin (Thermo Fisher Scientific, Waltham, MA). NCI-H1581 was cultured in RPMI 1640 (Biowest, Nuaillé, France) with 10% FBS and antibiotics, and NCI-H520 cells were cultured in RPMI 1640 (ATCC) with fetal bovine serum and antibiotic mix. The NIH 3T3 cell line was cultured in DMEM (Gibco, Waltham, MA) and supplemented with BS (bovine serum) and 100 U/mL penicillin and 100 μg/mL streptomycin. Cells were subcultured 2–3 times per week and grown at 37 °C with 5% CO_2_ and 90% humidity.

### Methods

#### PeptibodyC19 Preparation

A Mammalian expression system (CHO-S cells) was used to obtain recombinant peptibodyC19. The PeptideF coding sequence was cloned into the pLEV113 vector encoding the Fc domain and transfected into CHO-S cells. The production and purification were obtained as described previously by our group.^29,39,40^ Samples were collected during production and purification and visualized by western blotting using the anti-human IgG (Fc) antibody conjugated with HRP.

#### Surface Plasmon Resonance (SPR) Analysis

The FGFR1-peptibodyC19 interaction measurements were performed using Biacore 3000 instrument (GE Healthcare) at 25 °C, in PBS with 0.05% Tween 20, 0.1% BSA, 0.02% NaN_3_, pH 7.4. The extracellular domains of FGFR1 in Fc fusions (in 10 mM sodium acetate, pH 5.0) were immobilized on the CM4 sensor chip surface (GE Healthcare) at 1500 RU using an amine coupling protocol. To determine kinetic constants of the interaction between peptibodyC19 or peptibodyC19-PEG4vcMMAE and FGFR1, a set of dilutions of protein at the concentrations ranging from 20 to 320 nM were injected at a flow of 30 μL/min. The association and disassociation were monitored for 180 and 280 s, respectively. Between injections, 10 mM glycine (pH 1.5) was applied to regenerate the sensor chip surface. The kinetic data were fitted and analyzed with BIAevaluation 4.1 software using a 1:1 Langmuir binding model and the respective rate constants (*k*_on_ and *k*_off_) and *K*_d_ values were calculated.

#### Signaling Assay

The NIH-3T3 fibroblast cell line was used for signaling assay. Cells were seeded on a 6-well plate at 2 × 10^5^ cells per well in DMEM medium with 10% FBS and incubated overnight at 37 °C. The next day, the medium was exchanged to serum-free DMEM to starve the cells. After 16 h, peptibodyC19, FGF1, or Fc were added and incubated for 30 min in 37 °C. One well with untreated cells was used as a control. Cells were lysed using 2× Laemmli sample buffer, sonicated, and boiled. Proteins were separated by SDS-PAGE and analyzed by western blotting using antibodies against phosphorylated and total FGFR1 and Erk. Anti-gamma-tubulin antibody detection was used as a loading control.

#### Fibroblast Proliferation Assay

NIH-3T3 cells were seeded at 1 × 10^4^ cells per well on a 96-well plate in DMEM medium with 10% FBS and incubated overnight at 37 °C in 5% CO_2_. Then, the medium was exchanged to serum-free DMEM and cells were starved overnight. The next day, different amounts of peptibodyC19 and FGF1 with heparin were added to the wells and incubated for 48 h at 37 °C at 5% CO_2_. Wells without the addition of protein were used as controls. Alamar blue was added to the wells and incubated for 4 h. Cell viability was analyzed by measurement of fluorescence intensity at 590 nm (excitation at 560 nm) on an Infinite M1000 PRO plate reader (Tecan, Männedorf, Switzerland).

#### Fluorescence Microscopy

For colocalization assay, U2OS and U2OS-FGFR1 cell lines were used. Cells were plated in DMEM medium with 10% FBS on a 96-well plate at 1 × 10^4^ cells per well, transfected with CellLight Early Endosomes-RFP and incubated overnight at 37 °C in 5% CO_2_. The next day, cells were starved for 4 h and then incubated with peptibodyC19 (4 μg) or Fc domain (4 μg) on ice for 20 min. The plate was transferred to 37 °C for 30 min. Cells were fixed by incubation with 4% PFA for 15 min, washed with PBS, and permeabilized for 10 min with 0.1% Triton X-100. Next, the detergent was removed and wells were blocked with 2% BSA for 30 min. Fc-bearing proteins were visualized by incubation with Zenon Alexa Fluor 488 for 60 min and then the blocking agent was added and incubated for 5 min. Wells were washed three times with PBS and fixed with PFA as previously mentioned. After washing, the cells were incubated with NucBlue reagent for 5 min and washed again three times with PBS.

Colocalization was analyzed by wide-field fluorescence microscopy using a Zeiss Axio Observer Z1 fluorescence microscope with an LD-Plan-Neofluar 40/0.6 objective and Axiocam 503 (Zeiss, Germany). Images were processed with Zeiss ZEN 2.3 software (Zeiss, Germany) and Adobe Photoshop CS6 (Adobe, San Jose, CA, USA).

#### PeptibodyC19vcMMAE Conjugate Preparation

##### Conjugation with Cytotoxic Drug

To optimize the reduction of peptibodyC19, 20 μg of the protein was incubated at a concentration of 0.5 mg/mL in PBS pH 7.3 with TCEP—either 2 μM (10-fold excess over protein) or 1 mM, 1 mM EDTA, 1 M urea, and 5% glycerol in varying incubation time and temperature. After incubation, the peptibody was diluted to 0.2 mg/mL with buffer (PBS pH 7.3, 1 mM EDTA, 1 M urea, and 5% glycerol), and one of the cytotoxic drugs (vcMMAE, PEG_4_vcMMAE, PEG_27_vcMMAE, or PEG_27_vcMAY) was added and the mixture was incubated for 3 h in 15 °C.

Samples from the mixture after conjugation were collected, mixed with 2× Laemmli buffer, boiled at 95 °C for 10 min, and loaded on 12% SDS gel. In the case of visible precipitation, the sample was centrifuged (15,000 × *g*, 20 min) before mixing with Laemmli buffer. After electrophoresis, the number and height of bands were analyzed by Coomassie blue staining.

For scaling-up the reaction, 1 mg of peptibodyC19 and the following conditions were used—reduction: incubation for 1 h in RT, 1 mM TCEP, conjugation: 25 μL of PEG_4_-vcMMAE per 1 mg of peptibody. The efficiency of conjugation was analyzed with gel electrophoresis, as described above.

#### Purification of PeptibodyC19-PEG4vcMMAE

After conjugation, the reaction mixture was diluted five times with wash buffer (300 mM NaCl, 18 mM NaH_2_PO_4_, 33 mM Na_2_HPO_4_, 2 mM EDTA, 0.1% Tween 20, pH 7.5), loaded onto Protein A Sepharose equilibrated with wash buffer and washed with the same buffer. The conjugate was eluted with 100 mM triethylamine (TEA) and collected into tubes with 1 M Tris pH 7.2. Due to the peptibodyC19 pI, (6.1) standard elution with low pH resulted in partial protein precipitation; therefore, a high pH elution was performed. The buffer was exchanged to PBS pH 7.5 on a HiTrap Desalting column and the efficiency of conjugation and purification was analyzed by gel electrophoresis as described above.

The drug–protein ratio was determined spectrophotometrically.^41^ The absorbance of peptibodyC19-PEG_4_vcMMAE in PBS was measured at 248 and 280 nm. Extinction coefficients for MMAE (ε_MMAE_^248^ = 15,900 L/mol cm^–1^ and ε_MMAE_^280^ = 1500 L/mol cm^–1^) and peptibodyC19 (ε_pep_^248^ = 26,767 L/mol cm^–1^ and ε_pep_^280^ = 59,400 L/mol cm^–1^) were used.

#### Mass Spectrometry

MS spectra of peptibodyC19 and peptibodyC19-PEG_4_vcMMAE conjugates were acquired on a 4800 Plus MALDI-TOF/TOF (Applied Biosystem) with sinapic acid as the matrix.

#### Cytotoxicity Assay

HCC-95 (FGFR1-negative), NCI-H520 (FGFR1-positive), and NCI-H1581 (FGFR1-positive) cells were seeded on a 96-well plate at 5 × 10^3^/well in RPMI 1640 medium with 10% FBS and incubated overnight at 37 °C in 5% CO_2_. The next day, peptibodyC19-PEG_4_vcMMAE and peptibodyC19 at different concentrations (0.1–200 nM) were added to the cells and incubated for 96 h at 37 °C in 5% CO_2_. Wells with cells without a conjugate and with RPMI alone were used as negative and positive controls, respectively. After incubation, Alamar blue reagent was added to all wells and incubated for 4 h. The viability of cells was analyzed by measurement of fluorescence at excitation/emission of 560/590 nm on an Infinite M1000 PRO plate reader. Every experiment was performed in triplicates. EC_50_ values were calculated based on the Hill equation using Origin 7 software (Northampton, MA).

## Results

### PeptibodyC19 Efficiently Binds to FGFR1 In Vitro and In Vivo

**Figure 2:**
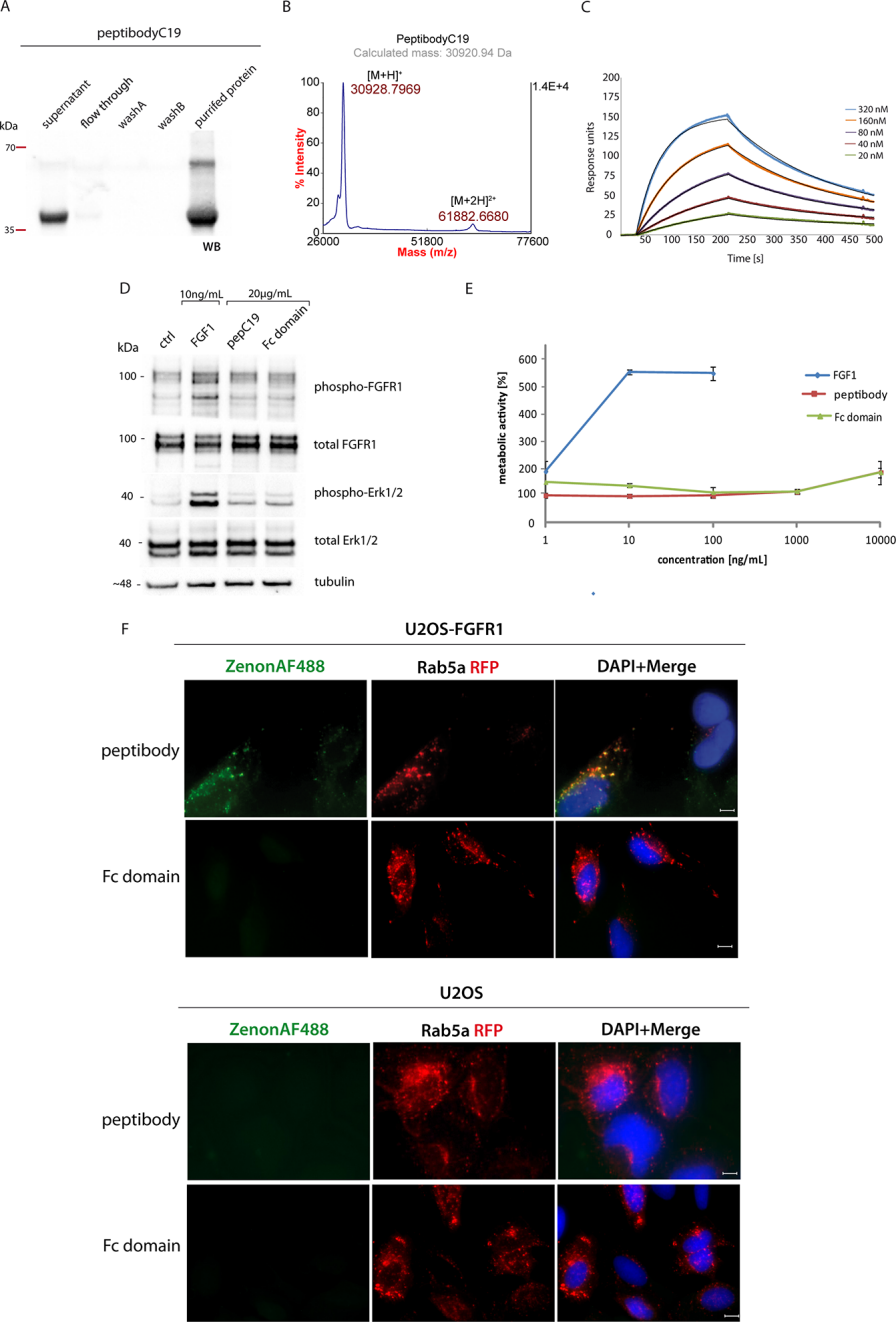
PeptibodyC19 binds FGFR1 and is internalized into FGFR1-expressing cells. (A) PeptibodyC19 was expressed in CHO cells and purified by ProteinA-affinity chromatography. Protein levels during the purification process were detected by western blot analysis with anti-Fc antibodies. The additional band visible on the western blot results from the presence of the peptibody dimer, due to the possibly incomplete sample reduction before electrophoresis. (B) Proper mass of purified protein was confirmed by mass spectrometry. (C) Kinetics of peptibodyC19 binding to FGFR1 measured by SPR. Titration with the peptibody in the concentration range from 20 to 320 nM allowed determination of *K*_d_ (87.7 nM), *k*_on_ (5.55 × 10^4^ s^–1^ M^–1^), and *k*_off_ (4.87 × 10^–3^ s^–1^) values. (D) Western blot analysis of signaling pathway activation by peptibodyC19. Ctrl – untreated cells. Antibodies against both phosphorylated and total FGFR1 and Erk1,2 were utilized. Anti-tubulin antibodies were used for loading control. (E) Internalization of peptibodyC19 into FGFR1-expressing cells evaluated with fluorescence microscopy. The Fc domain alone was used as a negative control. Fc-bearing proteins were labeled with ZenonAF 488. Early endosomes and nucleus were visualized by Rab5a RFP fusion and DAPI staining, respectively. The scale bar corresponds to 5 μm.

As mentioned above, one of the challenges with peptides is their lower target affinity compared with, e.g., monoclonal antibodies. Here, we have employed previously described peptide sequence binding FGFR1. Ballinger and colleagues identified by phage display a 26-amino acid peptide (C19) using the extracellular domain of FGFR1.^38^ This peptide shows affinity toward FGFR1 in vitro (*K*_d_ = 400 nM), which can be increased by peptide dimerization, e.g., in Fc-fusion (*K*_d_ = 90 nM). Effective binding was also observed in the fibroblast cell line model, making it a potential carrier molecule for FGFR1-targeted drug delivery. Importantly for its use as a targeted therapeutic, Fc-fusion was shown to lack mitogenic potential. We have fused the C19 peptide C-terminally to the Fc fragment from IgG1, forming a peptibody construct. PeptibodyC19 has been successfully overexpressed in CHO cells and purified by ProteinA-affinity chromatography, yielding ∼20 mg/1 L culture (Figure 2A). All steps of purification were monitored by polyacrylamide gel electrophoresis followed by Coomassie blue staining as well as western blot analysis with anti-Fc antibodies. The identity of the final purified product was also confirmed with MALDI-MS (Figure 2B).

To assay the interaction of peptibodyC19 with FGFR1 in vitro, we performed SPR analysis with the recombinant extracellular domain of FGFR1. PeptibodyC19 titration using the sensor with immobilized FGF receptors showed that the binding is dependent on peptibody concentration (Figure 2C). The calculated *K*_d_ value (87.7 nM) indicates a relatively high affinity of the peptibodyC19 or FGFR1 and is in good agreement with values reported previously (90 nM).^38^ The Fc domain, without any targeting peptide, did not show any significant binding to FGFR1 immobilized on the sensor, and peptibodyC19 did not show substantial binding to FGFR2 or FGFR3 immobilized on the sensor, confirming the specificity of its interaction with FGFR1 (Figure S1).

The binding of FGFs to FGFRs leads to signal transduction and modulation of various cellular processes. We have tested both the short-term response upon receptor stimulation, activation of its downstream signaling pathways, and the long-term, mitogenic response showing the proliferative potential of fibroblast cells.

To establish if the interaction of peptibodyC19 with FGFR1 triggers signaling pathway activation, NIH-3T3 fibroblast cells were incubated with the peptibody and phosphorylation levels of FGFR1 and downstream kinases Erk1,2 were assayed, with FGF1 and Fc used as positive and negative controls (Figure 2D). PeptibodyC19 and the Fc domain cause a slight increase in the phospho-Erk1,2 signal, though even concentrations as high as 20 μg/mL of peptibody do not cause signaling pathway activation comparable to much lower concentrations of FGF1. For the long-term response and estimation of peptibodyC19 mitogenic potential, a proliferation assay was performed on NIH-3T3 cells. PeptibodyC19 did not increase proliferation in concentrations up to 1 μg/mL (Figure 2E).

Ligand binding is one of the triggers for FGFR1 internalization; therefore, the level of peptibodyC19 internalized into FGFR1-expressing cells was tested with fluorescence microscopy. FGFR1-positive (U2OS-FGFR1) and FGFR1-negative (U2OS) cells were transfected with construct encoding Rab5a-RFP fusion for visualization of early endosomes, and peptibodyC19 or the Fc domain were labeled by Zenon Alexa Fluor 488. In FGFR1-overexpressing cells, colocalization of signals for peptibodies and endosomes was observed, whereas in U2OS cells lacking FGFR1, the internalization rate was negligible (Figure 2E). This indicates that peptibodyC19 is internalized in an FGFR1-dependent manner.

Overall, although peptibodyC19 was able to bind to the receptor and be internalized together with it, no significant activation of either signaling pathways or stimulation of cells’ proliferation rate was observed after treatment with the peptibody, demonstrating its potential value as a carrier for delivering the cytotoxic drug to cancer cells.

### PeptibodyC19 Thiol Conjugation Leads to Excessive Loading with the Drug, Which Can Be Optimized To Yield Functional Cytotoxic Conjugates

**Figure 3:**
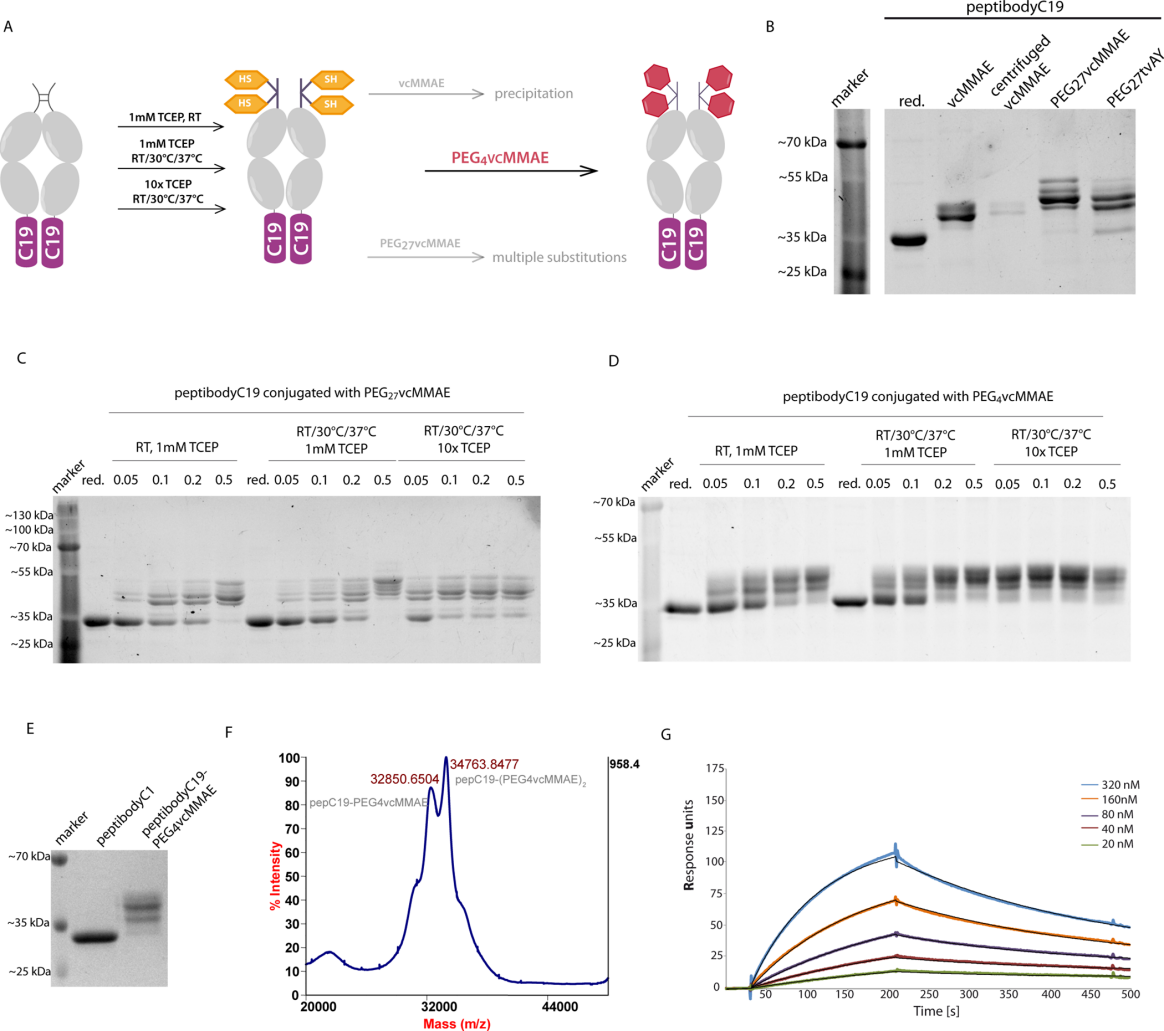
Optimization of conjugation reaction conditions allows obtaining functional cytotoxic conjugates. (A) Scheme of a standard antibody or Fc-bearing protein conjugation with maleimide-functionalized drugs. The desired outcome is drug molecules attached to cysteine residues within the Fc hinge region, which has been shown to not affect Fc domain properties. (B) Conjugation screening with different types of auristatin.(C) Optimization of conjugation reaction conditions for PEG_27_vcMMAE and (D) with PEG_4_vcMMAE. Values represent volume (μL) of drug per 20 μg of protein. Red – reduced protein. (E) Purification of peptibodyC19-PEG_4_vcMMAE on ProteinA-Sepharose. (F) Mass spectrometry analysis of peptibodyC19-PEG_4_vcMMAE by MALDI-TOF MS confirms modification with up to two drug molecules. (G) Kinetics of binding of peptibodyC19-PEG_4_vcMMAE with FGFR1 measured by SPR. Calculated *K*_d_ = 110 nM, *k*_on_ = 3.03 × 10^4^ s^–1^ M^–1^, *k*_off_ = 3.33 × 10^–3^ s^–1^.

Maleimide-based conjugation of MMAE to mAbs and Fc-bearing proteins utilizes cysteines in the Fc domain hinge region as attachment points for the drug (Figure 3A). However, peptibodyC19 contains, in addition, two cysteine residues in the targeting peptide sequence, which covalently modified with the drug could result in decreased affinity for the FGFR1 and less efficient delivery of the cytotoxic payload to cancer cells.

Because hydrophobic payloads decrease the stability of biomolecules and shorten the plasma half-life, and the size of the payload affects the degree of substitution to the biomolecule,^42^ we used four auristatin derivatives in the initial screen. As the most hydrophobic, we used monomethyl auristatin E. Next, we used more hydrophilic PEGylated derivatives of MMAE to increase the solubility of resulting conjugates. We utilized two PEG moieties differing in chain lengths (4 and 27 ether units), PEG_4_vcMMAE and PEG_27_vcMMAE, respectively. As the most hydrophilic and the biggest payload, we used hydrophilic auristatin Y decorated with PEG_27_ moieties (PEG_27_tvAY).^43,44^

For vcMMAE, most of the protein was lost due to precipitation. Reaction with PEG_27_vcMMAE resulted in a less abundant fraction of unconjugated peptibodies compared to the PEG_27_tvAY reaction, so it was further screened for optimal reduction conditions. The distinction between conjugates and unconjugated peptibodies was possible with polyacrylamide gel electrophoresis because attachment of the drug results in the sufficient shift in the mass of the protein to resolve them with SDS-PAGE.

Screening for optimal conjugation was performed for two versions of PEGylated MMAE, PEG_27_vcMMAE (Figure 3C) and PEG_4_vcMMAE (Figure 3D), differing in length of PEG chains attached to the drug. To manipulate the number of cysteines substituted in peptibodyC19, we have tested slightly varying reduction conditions, as this step is to determine how many thiol groups will be reduced and thus be available for chemical modification. TCEP, a reducing agent, was used at two different concentrations (either 2 μM, i.e., 10-fold excess over protein, or 1 mM) and two incubation temperatures were applied, ambient room temperature and a gradual change of temperature from room temperature to 37 °C, since at higher temperatures the reduction reaction proceeds faster and is more effective. PeptibodyC19 was incubated with a reducing agent in gradually increasing temperature to minimize the risk of protein unfolding.

Titration of peptibodyC19 with PEG_27_vcMMAE (Figure 3C) leads to a concentration-dependent increase in conjugation efficiency, with 2 μM TCEP being the more effective reductor for which even at the lowest tested drug concentrations a significant portion of the peptibody was conjugated. However, conditions in which only two drug molecules were attached to the protein showed at least 40% of peptibodyC19 still not conjugated. In the fully conjugated samples, populations of multiple modified peptibodyC19 were observed.

Taking into account that one of the reasons for this situation may be the relatively big size of the PEG molecule attached to the drug, we have decided to test also PEG_4_vcMMAE, with a shorter PEG chain. Indeed, it had better properties and did not yield as many multiple modified species. After reduction with 1 mM TCEP at room temperature and at sufficient PEG_4_vcMMAE concentrations yielded mostly double-substituted conjugates, with high efficiency and with little unconjugated protein left (Figure 3D). Nevertheless, increased reduction temperature leads to excessive substituted cysteines (or unwanted amine modifications). After optimizing reduction and conjugation conditions, the reaction was scaled up to conjugate 1 mg of protein with PEG_4_vcMMAE. For purification of peptibodyC19-PEG_4_vcMMAE, affinity chromatography with Protein A resin was used to remove any excessive free auristatin and to ensure proper folding of the Fc domain (Figure 3E). Mass spectrometry analysis showed that no unmodified peptibodyC19 is present in the sample, as well as that up to two drug molecules get covalently attached to the peptibody (Figure 3F). More detailed analysis with the use of IdeZ protease (specifically cleaving off the hinge region of the peptibody) and trypsin digest of peptibodyC19 and peptibodyC19-MMAE conjugates coupled with MS allowed us to pinpoint one MMAE modification site to the cysteine residue within the hinge region, (described in the Supporting Information, Figure S2 and Tables S1 and S2).

To confirm that modification with the cytotoxic drug did not alter FGF receptor binding properties significantly, the interaction of the purified conjugate with FGFR1 was then studied by SPR (Figure 3G). The conjugate showed kinetics and a *K*_d_ value (110 nM) comparable to unconjugated peptibodyC19 (87.7 nM).

### Evaluation of the Cytotoxic Effect of the PeptibodyC19-PEG-MMAE Conjugate

The crucial property of developed conjugates is their ability to selectively target cells expressing FGF receptor 1. We have chosen human lung cancer cell lines showing FGFR1 overexpression (NCI-H520 and NCI-H1581) and a cell line with physiological, low levels of FGFR1 (HCC95) as a control,^30,45^ and we used them for the assessment of peptibodyC19-PEG_4_vcMMAE cytotoxicity. One of the most important issues in the drug delivery system is the chosen selective and nontoxic carrier; therefore, FGFR1-positive and FGFR1-negative cells were treated with a range of either peptibodyC19 or peptibodyC19-PEG_4_vcMMAE concentrations.

**Figure 4:**
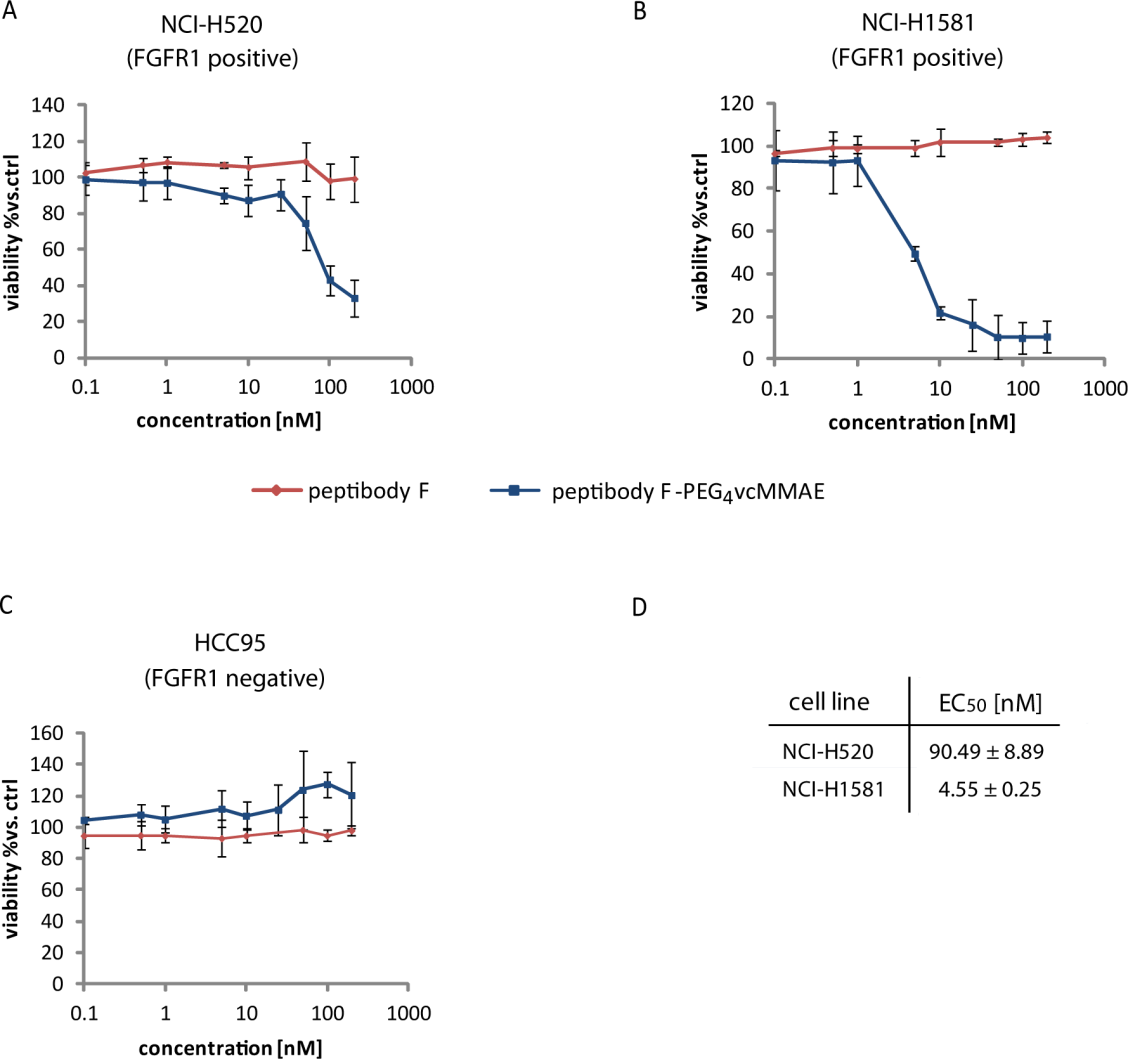
FGFR1-dependent cytotoxicity of peptibodyC19-PEG_4_MMAE on human lung cancer cell lines. FGFR1-positive cells, NCI-H520 (A) and NCI-H1581 (B), along with FGFR1-negative HCC95 cells (C) were treated with peptibodyC19-PEG_4_vcMMAE or peptibodyC19 alone for 96 h and their viability was estimated with the Alamar blue reagent. The error bars represent SEM from three independent experiments. (D) EC_50_ values for peptibodyC19-PEG_4_vcMMAE.

Both NCI-H520 and NCI-H1581 cells, FGFR1-positive, were sensitive for peptibodyC19-PEG_4_vcMMAE, while no toxicity was observed for FGFR1-negative HCC95cells (Figure 4A–C). We observed high, comparable with EC_50_ values observed for ADCs, cytotoxicity (EC_50_ at the nanomolar level) of peptibodyC19-PEG_4_vcMMAE to both FGFR1-positive cell lines. In the case of the NCI-H1581 cell line, we observed stronger susceptibility for conjugate compared with NCI-H520 cells (20-fold lower EC_50_ value). Noticeably, the pep-PEG_4_-vcMMAE conjugate caused the almost complete killing of the NCI-H1581 cell population (Figure 4B).

We did not observe a change in viability when the cells were treated with peptibodyC19 alone, both for cell lines with low levels of FGFR1 and receptor overexpression, confirming that FGFR binding does not lead to its activation and unwanted cell stimulation.

These results indicate that peptibodyC19-PEG_4_vcMMAE is specific for cells overexpressing FGFR1, which makes peptibodyC19 a promising candidate for a carrier for drug delivery.

## Discussion

Although a decrease in overall cancer mortality has been observed throughout the last decades, it remains unchanged for some types of cancer. For example, the mortality rate for lung cancer in 2015 was almost the same as in 1975,^46^ and it remains the most deadly and the second most common cancer after breast cancer.^47^ It is therefore not surprising that most clinical trials for cancer therapies focus on these two tumor types. Traditional therapies such as chemotherapy and radiotherapy are slowly being aided by and sometimes replaced by approaches aimed at reducing side effects and increasing specificity.^47^ Specific molecular markers of cancer cells can be identified and used as targets for inhibitors or blocking antibodies, derailing cancer cell functioning, or for the specific delivery of cytotoxic drugs (as in the case of antibody conjugates with cytotoxic drugs, ADCs).

To date, 12 ADCs have been approved by the FDA, and many more are in clinical trials.^48,49^ However, drug carriers are not limited to mAbs, and there are many other protein formats whose potential as targeting molecules is being investigated, mainly in basic research.^50^ Various cytotoxic molecules are used as a payload with a different mechanism of action. The most widely used are DNA-damaging agents such as duocarmycin, tubulin polymerization inhibitors like monomethyl auristatin E (MMAE) or mertansine, and inhibitors of topoisomerase II with doxorubicin as an example.^51^

ADCs approved by the FDA are aiming at different antigens, with HER-2, Nectin-4, and CD33 as examples.^48^ However, no treatment based on cytotoxic conjugates has yet been approved for cancers with FGFR1 overexpression. FGFR1 belongs to the tyrosine kinase receptor family, which is responsible for regulating many crucial processes of organism development and cellular metabolism.^52,53^ Overexpression of FGFR1 has been reported, among others, in breast and lung cancer, making it a promising target for therapy.^54^ Two FGFR-targeting ADCs have been studied in phase I clinical trials, LY3076226 (NCT02529553) and BAY1187982 (NCT02368951), specific for FGFR3 and FGFR2, respectively. In the preclinical studies, a tetravalent antibody, T-Fc, after conjugation with MMAE showed specific toxicity toward FGFR1 overexpressing cells.^55^ Protein formats consisting of parts of antibodies can also serve as carriers for the cytotoxic payload, e.g., scFv (single-chain variable fragment), Fab, or diabodies.^56−58^ scFv fusion with the Fc domain of IgG1 was developed to target FGFR1 and used as a vehicle to deliver MMAE to FGFR1-positive cancer cells,^59^ as well as a peptibody (peptide–Fc domain fusion) MMAE conjugate developed by us previously.^30^

Peptibodies combine the advantages of antibodies and targeting peptides making them promising candidates for targeted anticancer treatment. Other applications of this protein format include therapies for immune thrombocytopenic purpura and type 2 diabetes and inhibition of angiogenesis, with two FDA-approved drugs on the market.^60−62^ However, none of the peptibody–drug conjugates for cancer treatment has reached the market so far. The agents under development focus on various molecular targets. For example, recently reported R4Fu-Q65R-MMAE targets three different receptors, leucine-rich repeat containing G-protein-coupled receptors 4, 5, and 6 (LGR4–6), and shows promising results both in vitro and in vivo.^63^ LGR4–6, similar to FGFR1, are overexpressed in cancer cells. However, their overexpression is mainly found in gastrointestinal cancer, opposite to lung and breast cancers for FGFR1. The efficiency of R4Fu-Q65R-MMAE in vivo suggests the legitimacy of testing peptibodyC19-PEG_4_MMAE in animal models to further characterize its potential for anticancer treatment.

Alternatively, other FGFR-targeting molecules can be used as drug carriers. These include natural ligands for receptors or their altered versions with a high affinity for the target. We have shown that engineered variants of FGF1 conjugated with MMAE as well as FGF2-MMAE and FGF2-AY (auristatin Y) conjugates induce a cytotoxic effect in vitro, specifically in cells overexpressing FGFR1.^43,64,65^ We have also shown in a mouse model that FGF2 conjugated with PEGylated MMAE inhibits the growth of the tumor overexpressing FGFR1.^44^

Here, the FGFR1-targeting peptide was reformatted into a peptibody format and was produced, characterized, and conjugated with MMAE, a cytotoxic drug. Such conjugates were then characterized in terms of their toxicity in cell assays and showed specific toxicity against cancer cells overexpressing FGFR1. The targeting part of the peptibody was based on previously described peptide C19 screened by phage display and showing high affinity for FGFR1.^38^ Ballinger and colleagues described the agonistic action of the C19 peptide dimerized by the c-Jun leucine zipper; even though such construct showed superior FGFR1 binding properties, its mitogenic properties prevented its use as an anticancer agent. For this reason, an Fc-fusion of the C19 peptide presents a more suitable option as a nonstimulating, receptor binding drug carrier. Lack of peptibody proliferative activity has been confirmed by us, and its FGFR1-dependent internalization can be triggered without significant activation of receptor downstream signaling, a favorable feature for a molecule used as a vehicle in anticancer treatment.

PeptibodyC19 interaction with FGFR1 in vitro was studied by SPR. Kinetics showed concentration-dependent binding of peptibodyC19 to the receptor. The calculated *K*_d_ value (*K*_d_ = 87.7 nM) indicates strong interaction, placing peptibodyC19 between FGFR1 natural ligands—FGF1 (*K*_d_ = 136 nM) and FGF2 (*K*_d_ = 62 nM), which could enable peptibodyC19 to compete with FGFs in binding to the receptor.^66^ PeptibodyC19 affinity for FGFR1 was also stronger than that of the C19 peptide itself (*K*_d_ value 4.5 times lower), which demonstrates that reformatting peptides to the format of a peptibody can alter their binding properties.^38^ C19-Ig, a C19 peptide fused to the N-terminus of the IgG1 Fc fragment, presented by Ballinger et al. showed a similar affinity for FGFR1 (*K*_d_ = 90 nM), suggesting that protein dimerization driven by the Fc domain enhances avidity.

These favorable binding characteristics of peptibodyC19 make it a potential carrier molecule for cytotoxic drugs. We have decided to covalently fuse peptibodyC19 with MMAE, a highly cytotoxic drug used in ADC technology. The classical conjugation approach, based on maleimide, which has already been used in our group for the production of peptibody–drug conjugates,^29^ utilizes the thiol group of cysteines in the Fc domain. However, in peptibodyC19, cysteine residues are present also within the sequence of the targeting (C19) peptide. Attachment of MMAE to the fragment responsible for targeting FGFR1 could result in the decrease of affinity between the peptibody and the receptor. To eliminate the possibility of attaching a payload to the targeting peptide, cysteine residues within its sequence could be mutated to other amino acids, such as serine or alanine. However, these mutations could alter the affinity of peptibodyC19 or FGR1 and would require experimental verification.

For site-specific conjugation, unnatural amino acids can be utilized, as *p*-acetylphenylalanine (pAcF) and *p*-azidomethyl-*l*-phenylalanine (pAmF) incorporated into antibodies allowed for the attachment of the cytotoxic payload.^67^ Conjugation can also be driven by enzymes such as sortase A (SrtA), transglutaminases, and formylglycine-generating enzymes (FGE).^68^ Inteins, which are fragments of protein with endopeptidase activity, can be also used for conjugation. This approach has been used for the generation of bispecific antibodies and has many other applications in protein engineering.^69,70^ Other, less common methods utilizing addition to thiols have also been used for conjugation. These include disulfide rebridging, disulfide–thiol exchange, or reaction with sodium 4-((4-(cyanoethynyl)benzoyl)oxy)-2,3,5,6-tetrafluorobenzenesulfonate (CBTF) comprising 3-arylpropionitrile (APN) groups responsible for coupling with mAb.^71−73^ Nevertheless, in comparison with the abovementioned methods, the maleimide–thiol chemistry is characterized by the following features is simple, easy to control, and does not require the introduction of unnatural amino acids into the protein sequence.

As we proved here, manipulating time, temperature, the concentration of the reducing agent, and the peptibody–drug ratio allows for adjusting a number of substituted cysteines. In the first step, different forms of auristatin were screened. Hydrophobic properties of the MMAE decrease anticancer effectiveness of conjugates via stimulation of the aggregation process and then an acceleration of the plasma elimination process also increases the immune response directed against aggregated conjugates.^74^ During the conjugation reaction optimization, MMAE caused protein precipitation and was excluded from further screens. More hydrophilic PEGylated forms of AY and MMAE (PEG_27_-vcMMAE) did not result in peptibody aggregation and precipitation but generated conjugates with multiple substituted cysteines. This indicates possible attachment of the drug also to the targeting region of the peptibody, which could affect its binding to FGFR1. To reduce the number of substituted cysteines, further screening with various reduction conditions and drug amounts was performed. PEG_27_-vcMMAE was chosen for this step, and as for AY, more unconjugated protein was left in the previous reaction. Comparison of shorter and longer PEG chains attached to MMAE (PEG_4_-vcMMAE and PEG_27_-vcMMAE) resulted in the identification of reaction conditions allowing for substitution of the desired number of cysteines. After upscaling the conjugation and purification of peptibodyC19-PEG_4_-vcMMAE, its interaction with FGFR1 was studied by SPR, showing slightly weaker affinity (*K*_d_ = 110 nM) compared to unconjugated peptibodyC19, which may be a result of steric hindrance caused by MMAE and PEG chains. Despite reduced affinity, cytotoxicity assays showed a specificity of PepF-PEG_4_vcMMAE toward lung cancer cells overexpressing FGFR1, leaving cells lacking the receptor unaffected.

In summary, the results of this study demonstrate that peptibodyC19 can interact with FGFR1 and be specifically internalized into cells overexpressing the receptor. By manipulating the conditions for maleimide-based conjugation with auristatin derivatives, peptibody–drug conjugates with different numbers of substituted cysteines could be generated. The optimized reaction allowed for preparation of peptibodyC19-PEG_4_-vcMMAE, which showed selective cytotoxicity in cells with FGFR1 overexpression. Overall, the study provides evidence that after further testing, peptibodyC19 could make a potent candidate for drug carrier in cancer therapy.

## Supporting Information Available

SPR analysis of peptibodyC19 binding to other targets, MALDI-MS analysis of the peptibody hinge region after IdeZ protease cleavage, LC-ESI-MS analysis of tryptic fragments of peptibodyC19 and its conjugate (PDF)
