# Supplementary material for: Drug Conjugation via Maleimide–Thiol Chemistry Does Not Affect Targeting Properties of Cysteine-Containing Anti-FGFR1 Peptibodies
Source: Mol Pharm. 2022 Apr 7;19(5):1422–33. doi: 10.1021/acs.molpharmaceut.1c00946 (PMC9066409; doi:10.1021/acs.molpharmaceut.1c00946)
Supplement: Supplementary file 1 — mp1c00946_si_001.pdf [file mp1c00946_si_001.pdf]

Supplementary materials

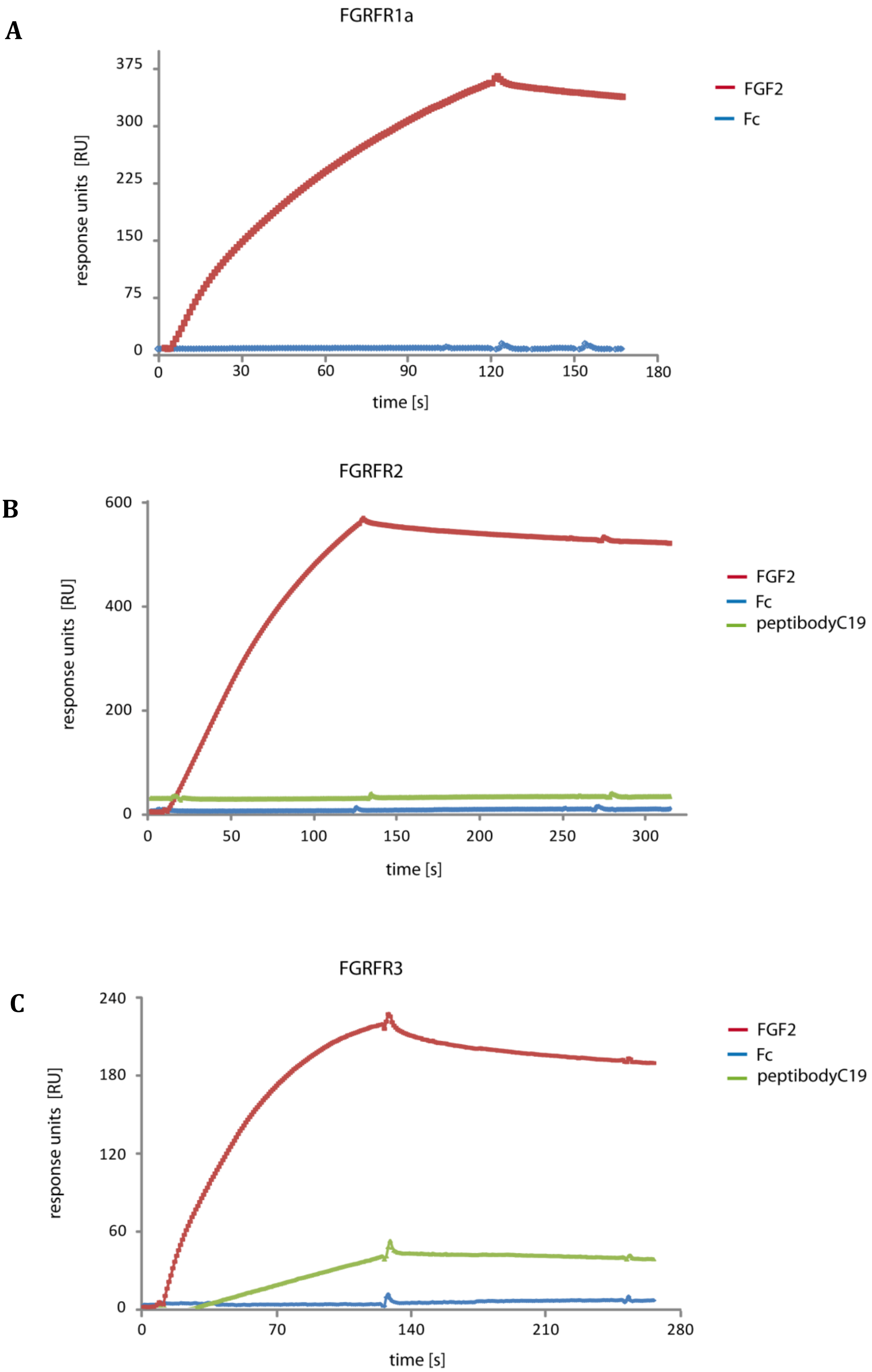

Figure S1. SPR analysis of peptibodyC19 binding to other targets. (A) Fc domain, without any targeting peptide, did not show any significant binding to FGFR1 immobilized on the sensor. (B-C) PeptibodyC19 showed no binding to FGFR2 and very little binding to FGFR3 immobilized on the sensor. FGF2 was used as a positive control to verify proper folding of immobilized receptors.

MALDI-MS analysis of peptibody hinge region after IdeZ protease cleavage

We performed proteolytic cleavage of peptibodyC19 and its conjugate. We used the IdeZ protease which specifically hydrolyzes the peptide bond right after the hinge region in the IgG sequence. Such cleavage results in an N-terminal peptide containing the hinge region and Fc domain.

In the case of peptibodyC19, we identified the peptide corresponding to the unmodified hinge region (Figure SA). In the case of the conjugate, we identified the peptide corresponding to the hinge region coupled with a single PEG4-vcMMAE molecule (Figure SB).

This result shows that one PEG4-vcMMAE molecule is coupled to Cys residue in the hinge region.

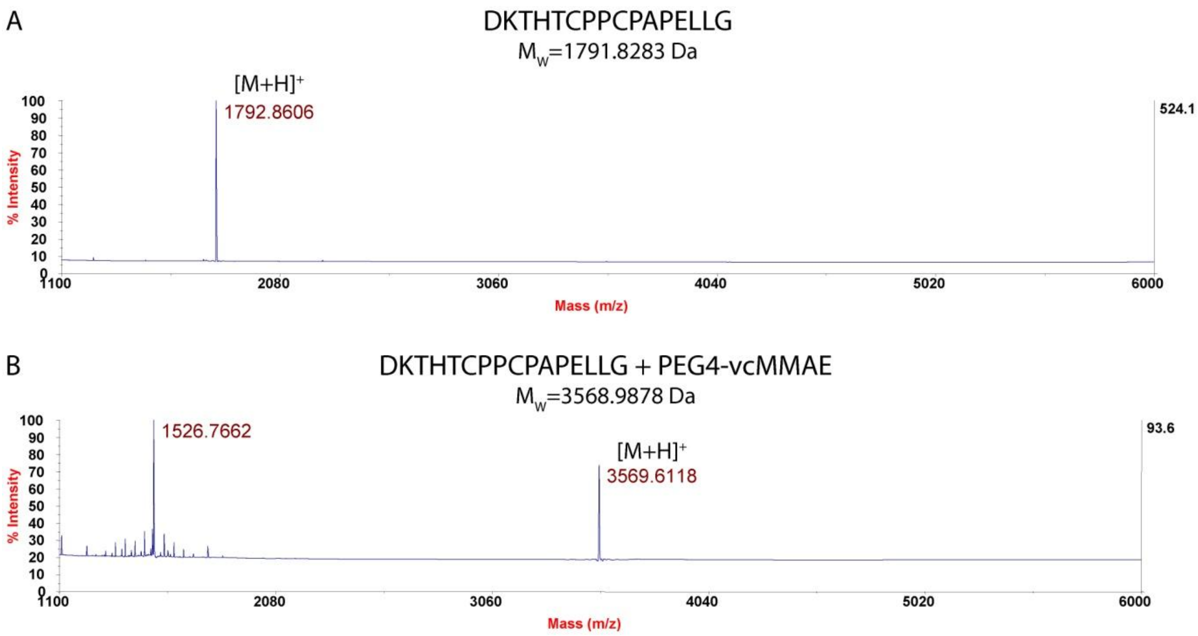

Figure S2. MALDI-MS analysis of peptibodyC19 (A) and peptiodyC19-PEG4-vcMMAE (B) after IdeZ protease cleavage.

LC-ESI-MS analysis of tryptic fragments of peptibody C19 and its conjugate

We performed tryptic digestion of the peptibodyC19 and its conjugate. Resulting tryptic fragments were analyzed by LC-ESI-MS (Table S1 and Table **S2**). In both cases, we identified the mass corresponding with AESGDDYCVLVFTDSAWTK peptide (2163.9538 Da with carbamidomethylated Cys, Cmpt B inTable S1 and Table **S2**) that is N-terminal fragment of peptide C19. This result shows that the second PEG4-vcMMAE molecule is not attached to the N-terminal region of the C19 peptide.

Table S1. Peak list after LC-ESI-MS analysis of the tryptic fragments of peptibodyC19.

Cmpt,	Mass	Molecule	Abund,	Abd,[%]	Std,Dev,
A	1873,9621	[M+H]+	38576	100	0,002121
**B**	**2163,9986**	**[M+H]+**	**34997**	**90,72**	**0,004051**
C	1930,9865	[M+H]+	10465	27,13	0,008601
D	1161,6476	[M+H]+	9188	23,82	0,00343
E	2139,0691	[M+H]+	5404	14,01	0,002426
F	2221,0213	[M+H]+	4885	12,66	0,003775
G	2544,1753	[M+H]+	3465	8,98	0,007457
H	4326,9794	[M+H]+	1030	2,67	0,006937
I	3746,9014	[M+H]+	1445	3,75	0,014639
J	788,4675	[M+H]+	2378	6,16	0,000551
K	2843,5017	[M+H]+	694	1,8	0,025836
L	2601,1949	[M+H]+	1101	2,85	0,003188
M	1677,8391	[M+H]+	1777	4,61	0,002363
N	1808,0457	[M+H]+	1513	3,92	0,002472
O	2196,0864	[M+H]+	1277	3,31	0,00271
P	838,5197	[M+H]+	1241	3,22	0,001828
Q	1865,0692	[M+H]+	1065	2,76	0,002303
R	4383,9987	[M+H]+	373	0,97	0,003053
S	4093,9631	[M+H]+	386	1	0,002709
T	1734,8536	[M+H]+	598	1,55	0,007144
U	2900,5092	[M+H]+	2596	6,73	0,019249
V	845,4891	[M+H]+	616	1,6	0,000288
W	1218,6698	[M+H]+	541	1,4	0,000458
X	6490,9578	[M+H]+	231	0,6	0,006721
Y	5619,8443	[M+H]+	110	0,29	0,004428
Z	895,542	[M+H]+	446	1,16	0,000791
a	2600,2214	[M+H]+	142	0,37	0,047061
b	948,9757	[M+H]+	405	1,05	0,000535

Table S2. Peak list after LC-ESI-MS analysis of the tryptic fragments of peptibodyC19-PEG4-vcMMAE.

Cmpt,	Mass	Molecule	Abund,	Abd,[%]	Std,Dev,
A	1873,9652	[M+H]+	11663	100	0,001764
**B**	**2164,0003**	**[M+H]+**	**2906**	**24,91**	**0,002708**
C	1161,65	[M+H]+	2164	18,55	0,00322
D	2544,1824	[M+H]+	1228	10,53	0,007378
E	2139,0727	[M+H]+	1433	12,28	0,001748
F	3746,9096	[M+H]+	248	2,13	0,003289
G	1677,8411	[M+H]+	473	4,05	0,000683
H	825,8463	[M+H]+	460	3,95	0,001727
I	1930,9876	[M+H]+	397	3,41	0,002345
J	413,4305	[M+H]+	431	3,69	0,000383
K	1808,048	[M+H]+	388	3,32	0,001412
L	788,4699	[M+H]+	373	3,2	0,000518
M	807,8352	[M+H]+	308	2,64	0,000171
N	1895,9457	[M+H]+	256	2,19	0,000634
O	838,5224	[M+H]+	235	2,01	0,001818
P	2601,2013	[M+H]+	120	1,03	0,001852
Q	6717,6564	[M+H]+	112	0,96	0,002749
R	5598,8927	[M+H]+	138	1,19	0,134632

Supplementary methods

IdeZ cleavage of peptibodyC19 and peptibodyC19-conjugate

PeptibodyC19 and peptibodyC19-conjugate was digested with IdeZ (IgG-specific protease) according to the manufactures' protocol (New England, BioLabs, Ipswich, USA). We extended incubation time from 30 minutes to 1 hour at 37°C.

Trypsin digestion in solution

Preparation of samples for LC-ESI-MS analysis was based on theprotocol from Leize-Wagner group from 2016 (Said et al., 2016). PeptibodyC19 and its conjugate were heated to 40°C for 10 minutes. Next, 1 mM TCEP was added and incubated for 10 minutes at 80°C and cooled down to room temperature. Samples were alkylated with 50mM iodoacetamide at room temperature for 30 minutes. Acetonitryle to final concentration of 10% and 1μg of trypsin in Tris-HCl pH 8.0 with $CaCl_2$ were added and incubated for 3 hours at room temperature, and another 1μg of trypsin was added afterwards. The reaction was continued overnight at 37°C. The samples were reduced for the second time by addition of 1 mM TCEP and incubated at 56°C for 45 minutes. After that, 40% isopropanol and 1% formic acid were added and samples were incubated at room temperature for 2h.

LC-ESI-MS analysis of peptibody C19 and its conjugate fragments

(20 μL) of digests were loaded via autosampler onto a C18 column enclosed in a thermostatted column oven set to 60 °C (Vanquish Flex UHPLC, Thermo Scientific). Samples were held at 12 °C while queued for injection. The chromatographic method was initiated with 5% ACN with 0.1% formic acid. After a 5 min equilibration, peptides were eluted over a 50 min gradient in which ACN content rose to reach 70%. Prior to the next sample injection, the column was washed for 10 min with 90% ACN, then equilibrated at 5% for 15 min. The eluate was diverted to waste for the first 15 min and final 20 min of the run.

Peptides eluting from the chromatography column were analyzed by UV absorption at 220, 248, 280 nm followed by mass spectrometry on the Bruker Compact QTOF (Bruker Daltonics) operated in the positive ionization mode and calibrated with ESI Tuning Mix (Agilent Technologies). Sample solutions were introduced into the MS at a rate of 250 µl/min. Settings: scan range = 400–3000 m/z; nebulizer = 1.8 bar; dry gas = 220°C and 8.0 l/min; capillary = 4500 V; end plate offset = 500 V; hexapole RF = 300 Vpp. Mass range was 350 to 4000 m/z. The spectra were averaged over 15–60 min collection time and deconvoluted using the Bruker Compass Data Analysis software package.

References:

Said, N., Gahoual, R., Kuhn, L., Beck, A., François, Y. N., & Leize-Wagner, E. (2016). Structural characterization of antibody drug conjugate by a combination of intact, middle-up and bottom-up techniques using sheathless capillary electrophoresis - Tandem mass spectrometry as nanoESI infusion platform and separation method. *Analytica Chimica Acta*, *918*, 50–59. https://doi.org/10.1016/j.aca.2016.03.006
